# Hyponatremia and Abdominal Pain: A Case Report of Acute Hepatic Porphyria

**DOI:** 10.7759/cureus.101733

**Published:** 2026-01-17

**Authors:** Marta Roldão, Antony Soares Dionísio, Joana Duarte, Andreia Salgadinho Machado, Marta Anastácio

**Affiliations:** 1 Internal Medicine, Centro Hospitalar de Lisboa Ocidental - Hospital São Francisco Xavier, Lisbon, PRT; 2 Intermediate Medical Care Unit, Internal Medicine, Centro Hospitalar de Lisboa Ocidental - Hospital São Francisco Xavier, Lisbon, PRT

**Keywords:** abdominal pain, hemin therapy, hyponatremia, porphobilinogen, porphyria

## Abstract

Acute hepatic porphyrias (AHPs) are rare metabolic disorders of heme biosynthesis that present with neurovisceral symptoms such as abdominal pain, vomiting, and hyponatremia. Diagnosis is frequently delayed due to the nonspecific nature of the symptoms and insufficient awareness of the disease.

A 23-year-old woman presented with diffuse abdominal pain, vomiting, myalgias, and severe hyponatremia (Na+ 104 mmol/L). Initial workup during hospitalization was inconclusive. Given the neurovisceral presentation and features of the syndrome of inappropriate antidiuretic hormone secretion (SIADH), including hypoosmolar serum and inappropriately concentrated urine, acute porphyria was suspected. Screening with a positive Hoesch test was performed, and urine samples for porphyrin precursor analysis were collected before discharge. After discharge, results confirmed markedly elevated urinary porphobilinogen and delta-aminolevulinic acid (ALA), confirming the diagnosis of acute hepatic porphyria. She was followed up in internal medicine and metabolic diseases. Despite preventive measures, she experienced a new acute attack requiring intravenous hemin.

This case highlights the diagnostic challenge of acute porphyria. Hyponatremia, often due to SIADH, and elevated creatine kinase (CK) levels can be key biochemical clues. Early suspicion and targeted testing are essential for diagnosis and timely therapy. Acute hepatic porphyria should be considered in young patients with unexplained abdominal pain, vomiting, and severe hyponatremia. Early recognition, prompt biochemical testing, and appropriate management are key to improving outcomes.

## Introduction

Acute hepatic porphyrias (AHPs) are inherited disorders characterized by partial enzyme deficiencies in the heme biosynthesis pathway, leading to accumulation of neurotoxic intermediates such as delta-aminolevulinic acid (ALA) and porphobilinogen (PBG) [1]. These include acute intermittent porphyria (AIP), hereditary coproporphyria (HCP), and variegate porphyria (VP).

The main clinical presentations include severe abdominal pain, vomiting, neuropsychiatric symptoms, and autonomic dysfunction. Hyponatremia, usually secondary to syndrome of inappropriate antidiuretic hormone secretion (SIADH), is a common and serious complication [2-4]. Recent guidelines emphasize the importance of considering AHP in the differential diagnosis of young patients presenting with neurovisceral symptoms, especially when accompanied by unexplained hyponatremia [5,6].

Diagnosis is often delayed due to the nonspecific nature of the symptoms, which may mimic common conditions such as acute gastroenteritis. Early biochemical testing during attacks, including measurement of urinary PBG and ALA, is critical [5,7]. Screening tests such as the Hoesch or Watson-Schwartz tests can guide early investigation while awaiting confirmatory assays. Intravenous hemin is the treatment of choice in moderate to severe attacks, while high carbohydrate intake, usually glucose, and supportive care are important adjuncts in the management of chronic disease [8,9]. More recently, givosiran, an RNA interference (RNAi) therapeutic targeting hepatic ALA synthase 1 mRNA, has emerged as a novel disease-modifying treatment in recurrent attacks [10-12].

## Case presentation

A 23-year-old woman with no relevant past medical history, except for an anxiety disorder without pharmacologic treatment, presented to the emergency department with a six-day history of diffuse abdominal pain, repeated vomiting, myalgias, and episodes of lipothymia. She reported no alcohol or drug use and no recent medication changes and had no known family history of porphyria.

Physical examination showed diffuse abdominal tenderness without peritoneal signs. Initial laboratory tests revealed microcytic hypochromic anemia (Hb 9.0 g/dL), hyponatremia (126 mmol/L), and hypochloremia (88 mmol/L). Liver and renal function were normal. She was initially treated for presumed bacterial gastroenteritis with fluids and empiric metronidazole and was discharged home.

The following day, she returned with persistent symptoms, without seizures or focal neurological deficits. On clinical examination, the patient was euvolemic, with no signs of dehydration, orthostatic hypotension, peripheral edema, or ascites. There was no history of recent diuretic use. Blood tests revealed worsening hyponatremia (Na+ 103 mmol/L), low serum osmolality (213 mOsm/kg), and inappropriately concentrated urine (urinary sodium 34 mmol/L, urine osmolality 307 mOsm/kg), elevated AST (160 U/L), ALT (44 U/L), markedly raised CK (4012 U/L), and hypochloremia (68 mmol/L), as shown in Table 1. Renal and thyroid function remained normal, and inflammatory markers and viral serologies were negative. Abdominopelvic CT revealed only aerocolia of the small bowel.

**Table 1:** Laboratory Assesment

Parameter	Normal Range	Laboratory Assessment		
		1st Emergency Department	2nd Emergency Department	Discharge
Hemoglobin	12.0–15.0 g/dL	10.3 g/dL	9.8 g/dL	10.3 g/dL
Mean corpuscular volume (MCV)	80.0–96.1 fL	69.4 fL	67.2 fL	78 fL
Mean hemoglobin concentration (MCH)	27.3–33.7 pg	23.4 pg	23.2 pg	25.7 pg
Leukocytes	4.0–10.0 × 10^9^/L	9.9 × 10^9^/L	8.9 × 10^9^/L	5.3 × 10^9^/L
Creatinine	0.50–0.90 mg/dL	0.90 mg/dL	0.80 mg/dL	0.90 mg/dL
Urea	13–43 mg/dL	27 mg/dL	109 mg/dL	110 mg/dL
Sodium	136–145 mmol/L	126 mmol/L	104 mmol/L	135 mmol/L
Potassium	3.5–5.1 mmol/L	4.1 mmol/L	4.62 mmol/L	4.72 mmol/L
Chloride	98–107 mmol/L	88 mmol/L	68 mmol/L	97 mmol/L
C–reactive protein (CRP)	<0.50 mg/dL	<0.10 mg/dL	0.10 mg/dL	<0.10 mg/dL
Aspartate aminotransferase (AST)	<32 U/L	48 U/L	160 U/L	25 U/L
Alanine aminotransferase (ALT)	<33 U/L	25 U/L	44 U/L	41 U/L
Creatine kinase (CK)	<170 U/L	-	4012 U/L	41 U/L
Thyroid-stimulating hormone (TSH)	0.27–4.20 UI/mL	-	1.89 UI/mL	-
EBV, Ac anti-VCA IgM	-	-	Negative	-
HIV 1/2 antibodies	-	-	Negative	-
Urinary osmolarity	300–900 mOsm/L	-	307mOsm/L	-
Urinary sodium	-	-	34 mmol/L	-
Hoesch screening test	-	-	-	Positive
Urinary porphobilinogen (PBG)	<10 µmol/L	-	-	243 µmol/L
PBG/creatinine ratio	<0.89 µmol/mmol	-	-	21.53 µmol/mmol
Delta-aminolevulinic acid (ALA)	<50 µmol/L	-	-	152.31 µmol/L
ALA/creatinine ratio	<2.6 µmol/mmol	-	-	13.49 µmol/mmol

She was admitted to the Intensive Care Unit for continuous monitoring and further investigation. Treatment consisted of 3% hypertonic saline administered as a continuous infusion. No neurological deterioration occurred, and there were no clinical or radiological features suggestive of osmotic demyelination syndrome. Given the combination of abdominal pain, severe hyponatremia, and marked creatine kinase elevation, several differential diagnoses were systematically considered. Adrenal insufficiency was excluded based on normal cortisol and ACTH levels. Hypovolemic hyponatremia secondary to gastrointestinal losses was considered; however, the patient was clinically euvolemic, and laboratory findings were not consistent with this diagnosis. Rhabdomyolysis related to exertion, heat exposure, or seizures was also considered, but there was no history of intense physical activity, hyperthermia, trauma, or seizure activity. The elevation of creatine kinase associated with a disproportionate increase in AST relative to ALT supported a muscular rather than hepatic source of transaminase elevation, consistent with muscle involvement in the clinical presentation. Drug-induced SIADH, including exposure to over-the-counter medications, was carefully reviewed and excluded. Pregnancy testing was performed and was negative. Thyroid function tests, celiac serologies, and echocardiography were normal. Thoracoabdominopelvic CT excluded malignancy and neuroendocrine tumors.

The clinical picture raised suspicion of acute hepatic porphyria, prompting a Hoesch screening test, which yielded a positive result. A urine sample was also collected for porphyrin precursor analysis, including PBG and ALA. The patient gradually improved and was discharged after 10 days, with follow-up scheduled in Internal Medicine and Metabolic Diseases.

Porphyrin precursor results obtained after discharge confirmed the diagnosis, with urinary porphobilinogen (PBG) of 243 µmol/L, a PBG to creatinine ratio of 21.53 µmol/mmol, delta-aminolevulinic acid (ALA) of 152.31 µmol/L, and an ALA to creatinine ratio of 13.49 µmol/mmol.

She was advised to avoid potential triggers, including barbiturates, certain antiepileptics such as phenytoin and carbamazepine, rifampicin, sulfonamide antibiotics, estrogen-containing therapies, and alcohol, and was followed up in the outpatient setting. Despite preventive measures, she experienced a new neurovisceral crisis requiring hospitalization and treatment with intravenous hemin, with subsequent clinical improvement and discharge. Long-term follow-up information was unavailable, as the patient was lost to follow-up.

## Discussion

This case highlights the diagnostic challenges of acute hepatic porphyria, a disease often overlooked due to its nonspecific presenting symptoms. Hyponatremia, secondary to SIADH, was remarkable in this particular patient and remains a hallmark of acute attacks [2,4,11]. Recent expert recommendations support early use of vasopressin receptor antagonists (e.g., tolvaptan) in refractory hyponatremia in porphyria, although data remain limited [11]. Elevated CK levels suggested muscle involvement or early rhabdomyolysis, a less frequently described but recognized complication in severe attacks. Awareness of neuromuscular features, including proximal muscle weakness, can help differentiate AHP from other causes of abdominal pain and electrolyte imbalance [5,10].

The Hoesch test, a rapid screening method, played a key role in raising clinical suspicion. Confirmatory biochemical analysis demonstrated markedly elevated urinary PBG and ALA, which are the diagnostic gold standards during symptomatic periods [5-7].

Early diagnosis is essential for treatment initiation. Intravenous hemin effectively suppresses ALA synthase activity, halting the accumulation of toxic metabolites and reducing symptom severity and recurrence risk [8,9]. For patients with recurrent attacks (≥4/year), givosiran has been shown to significantly reduce the frequency of attacks, lower hemin use, and improve quality of life [9,10]. Patient education on avoiding precipitating factors and close follow-up are crucial to disease management. The interval between sample collection during hospitalization and result confirmation after discharge illustrates a common challenge in the management of this disease.

This case highlights the need for increased clinical awareness of this disease to avoid diagnostic delays and complications, and to prompt the initiation of targeted treatment.

## Conclusions

Acute hepatic porphyrias should be included in the differential diagnosis of young patients with unexplained abdominal pain, persistent vomiting, severe hyponatremia, and neuromuscular symptoms. The presence of SIADH and elevated CK may serve as important clues. Rapid screening tests, such as the Hoesch test, alongside definitive biochemical assays (urinary PBG and ALA), are crucial for diagnosis, even when results become available only after discharge. Early identification and initiation of treatment with intravenous hemin improve clinical outcomes and reduce morbidity. In cases of recurrent attacks, givosiran represents a promising disease-modifying therapy. Increased clinical suspicion and compliance with current guidelines are vital for improving long-term outcomes.
